# *Klebsiella pneumoniae* induced ferroptosis by inhibition of the Nrf2/xCT/GPX4 pathway in bovine mastitis: *In vivo* and *in vitro*

**DOI:** 10.1080/21505594.2025.2600142

**Published:** 2025-12-04

**Authors:** Peng Mao, Zhihao Wang, Changning Yuan, Kangjun Liu, Long Guo, Junsheng Dong, Luying Cui, Jianji Li, Guoqiang Zhu, Xia Meng, Hongyun Liu, Lili Zhang, Ran Wang, Heng Wang

**Affiliations:** a College of Veterinary Medicine, Jiangsu Co-Innovation Center for Prevention and Control of Important Animal Infectious Diseases and Zoonoses, Yangzhou University, Yangzhou, China; b International Research Laboratory of Prevention and Control of Important Animal Infectious Diseases and Zoonotic Diseases of Jiangsu Higher Education Institutions, Yangzhou University, Yangzhou, China; c Joint International Research Laboratory of Agriculture and Agri-Product Safety of the Ministry of Education, Yangzhou University, Yangzhou, China; d Institute of Dairy Science, Zhejiang University, Hangzhou, China; e Institute of Food Safety and Nutrition, Jiangsu Academy of Agricultural Sciences, Nanjing, China

**Keywords:** Ferroptosis, *klebsiella pneumoniae*, bovine mastitis, ferrostatin-1, bovine mammary epithelial cells

## Abstract

*Klebsiella pneumoniae* (*K. pneumoniae*), a prominent causative agent of mastitis in dairy cattle, remains enigmatic in its pathogenic mechanisms. This study aimed to reveal the effects of *K. pneumoniae* on mammary glands via the induction of ferroptosis, as well as the protective role of Ferrostatin-1 (Fer-1) against this pathogen-mediated damage in bovine mammary epithelial cells (BMECs). Holstein cows were used to establish an intramammary infection model of *K. pneumoniae*. *In vitro*, primary BMECs were treated with 10 μM Fer-1 and *K. pneumoniae* alone or in combination. The results showed that mammary glands infected with *K. pneumoniae* exhibited increased transcriptional levels of *interleukin* (*IL*)*-1β*, *IL-6*, *IL-8*, and *tumor necrosis factor-α* (*TNF-α*). Concurrently, significant elevations in iron, 4-hydroxynonenal, and reactive oxygen species (ROS) levels were observed. Conversely, *K. pneumoniae* infection downregulated nuclear factor erythroid 2-related factor 2 (Nrf2), cystine/glutamate antiporter (xCT), glutathione peroxidase 4 (GPX4), and glutathione levels. Following *K. pneumoniae* invasion, intracellular Fe^2+^ and lipid ROS accumulated in the BMECs, impeding activation of Nrf2/xCT/GPX4 signal transduction. Additionally, Fer-1 facilitated the nuclear translocation of Nrf2 protein, upregulating the protein levels of Nrf2/xCT/GPX4 while downregulation transcriptional levels of *IL-1β*, *IL-6*, *IL-8*, and *TNF-α*. In conclusion, Fer-1 alleviates *K. pneumoniae*-induced inflammatory factor activation and ferroptosis in BMECs via upregulation of the Nrf2/xCT/GPX4 pathway, supporting ferroptosis inhibition holds promise as a feasible therapeutic agent for the control of mastitis.

## Introduction

Ferroptosis is an iron-dependent programmed cell death that can be triggered by inhibiting the cystine/glutamate antiporter (xCT) or through direct suppression of glutathione peroxidase 4 (GPX4). GPX4 utilizes glutathione (GSH) as a cofactor to reduce lipid peroxides, thereby maintaining membrane integrity [1]. The morphological hallmarks of ferroptosis include small mitochondria with condensed membrane densities, often accompanied by diminution or disappearance of mitochondrial cristae, and a notable disruption of the outer mitochondrial membrane [2]. Recent researches have highlighted the significant association between ferroptosis and infection, particularly bacterial infections [3–6]. Oxidative stress is a key pathological feature of bacterial infections [7]. When the cellular antioxidant system becomes dysregulated, ferrous ion (Fe^2+^) accumulation promotes the Fenton reaction, leading to excessive production of reactive oxygen species (ROS), particularly hydroxyl radicals [8]. ROS have the potential to interact with polyunsaturated fatty acids present within the cellular membrane, leading to the destabilization of the lipid bilayer, disruption of the membrane, and eventual initiation of ferroptosis. NFE2L2 encodes nuclear factor erythroid 2-related factor 2 (Nrf2), which plays a pivotal role in maintaining redox balance and regulating inflammation [9]. Upon activation, Nrf2 migrates to the nucleus, initiating the transcription of antioxidants, such as xCT [10] and GPX4 [11,12], which increases intracellular GSH levels and reduces ROS, thereby protecting cells from ferroptosis.

*Klebsiella pneumoniae* (*K. pneumoniae*) is a prevalent mastitis pathogen that has caused substantial economic losses globally in recent years [13]. *K. pneumoniae* isolated from milk harbors a diverse array of virulence and drug-resistance genes, enabling its rapid adherence to and invasion of bovine mammary epithelial cells (BMECs) [14]. *K. pneumoniae* invasion triggered cell damage and prompted BMECs to produce a profusion of inflammatory mediators, inclusive of interleukin-1β (IL-1β), interleukin-6 (IL-6), interleukin-10, and tumor necrosis factor-α (TNF-α) [15]. Furthermore, *K. pneumoniae* provokes extensive damage and dysfunction within the mitochondria of BMECs [16]. A previous study showed that *K. pneumoniae* causes oxidative stress and inhibited the activation of Nrf2 in BMECs [17]. These characteristics of *K. pneumoniae*-induced mastitis are strikingly similar to those of ferroptosis. Notably, previous studies have reported a significant presence of ferric iron and ROS in the mammary gland tissue of dairy cows with clinical mastitis [18]. Therefore, modulating Nrf2 activity and its downstream targets may offer new therapeutic potential by enhancing cellular antioxidant defenses and reducing oxidative stress-induced ferroptosis during *K. pneumoniae* infection.

Ferrostatin-1 (Fer-1), an arylalkylamine, is a pioneering synthetic radical-trapping antioxidant that inhibit ferroptosis [19]. Recently, the therapeutic potential of Fer-1 has been shown to extend to infectious diseases, such as sepsis and fowl adenovirus infection [20,21]. Studies have demonstrated that Fer-1 enhances Nrf2 expression and mitigates LPS-induced ferroptosis in vascular endothelial cells [22]. Furthermore, treatment with Fer-1 reduced the expression levels and distribution patterns of inflammatory cytokines, including IL-1β, IL-6, interleukin-8 (IL-8), and TNF-α, in a rat model of subarachnoid hemorrhage [23]. Despite these advancements, the role of Fer-1 in treating dairy cow mastitis, particularly in the context of *K. pneumoniae* infections, and its underlying molecular mechanisms remain unclear.

This study aimed to elucidate the relationship between ferroptosis and *K. pneumoniae*-induced mastitis in dairy cows. Additionally, we investigated the effect of Fer-1 on the Nrf2/xCT/GPX4 pathway to clarify its protective role against *K. pneumoniae*-mediated injury in BMECs. The findings of this study hold the potential to inform novel control strategies for managing *K. pneumoniae* mastitis.

## Materials and methods

### Reagents

The fetal bovine serum was purchased from LONSERA (Shuangru Biotechnology, Suzhou, China); Dulbecco’s modified eagle’s medium/F12 (DMEM/F12), collagenase (C2-BIOC), Hoechst (B2261), ferrostatin-1 (SML0583) and polyvinylidene difluoride (PVDF) were purchased from Sigma-Aldrich (St. Louis, USA); anti-4-hydroxynonenal (4-HNE) antibody (MAB3249, 1:250 dilution) was provided by R&D Systems (Minneapolis, USA); the anti-cytokeratin 18 antibody (ab233913, 1:100 dilution) was purchased from Abcam (Cambridge, USA); the anti-GAPDH antibody (#5174, 1:1000 dilution) and anti-xCT antibody (#12691, 1:1000 dilution) were purchased from Cell Signaling Technology (Danvers, USA); the anti-Nrf2 antibody (AF0639, 1:250 dilution for immunofluorescence, 1:1000 dilution for Western Blot) was purchased from Affinity Biosciences (Liyang, China); anti-GPX4 (sc-50497, 1:1000 dilution) and anti-prostaglandin-endoperoxide synthase 2 (PTGS2, sc-376861, 1:1000 dilution) antibodies were purchased from Santa Cruz Biotechnology (Santa Cruz, USA); Thermo Fisher Scientific (Waltham, USA) was supplied with Alexa Fluor™ Plus 488 (#A32731, 1:200 dilution) and goat anti-rabbit IgG (H+L) secondary antibody, HRP (#31460, 1:10000 dilution); ImmunoWay (Plano, USA) provided 4–6-diamidino-2-phenylindole (DAPI, YS0014); Beyotime Biotechnology (Shanghai, China) supplied the bicinchoninic acid (BCA) protein assay kit and Triton X-100 (P0096).

### Klebsiella pneumoniae culture

Hypermucoviscous *K. pneumoniae* (KPTP11), isolated from a milk sample from bovine mastitis [24–26], was used in this experiment. The sequence type (ST) of *K. pneumoniae* is ST35, and the NCBI accession is SAMN43087377. *K. pneumoniae* was cultured on Luria-Bertani (LB) agar at 37°C. The separate colonies were placed in 10 mL liquid LB and cultured in a shaker (THZ-312, Shanghai Jinghong Experimental Equipment Co., Ltd., Shanghai, China) at 120 rpm and 37°C overnight. Bacterial growth was monitored by measuring optical density at 600 nm (Epoch 2, BioTek Instruments, Inc., Winooski, USA).

### Animals and treatment

The experimental protocol was approved by the Animal Care and Ethics Committee of the Yangzhou University (approval ID: 202,205,130). The experiment was conducted at a dairy farm of the Yangzhou University. As the institution was the owner of the animals, a written informed consent for their participation in this study was provided by the university administration within the ethical approval framework. Six Holstein cows were rigorously matched for parity (an average parity of 2.5) and milk production (23.1 ± 2.8 kg/day) consistency during late lactation (240 ± 6 days postpartum), while excluding animals with > 5% daily yield fluctuation or clinical history of metabolic disorders within 30 days. A professional veterinarian confirmed that all dairy cows possessed four functional and healthy udder quarters with no treatment history for one month prior to the trial and were free of mycoplasma contamination. The cows were provided ad libitum access to fresh water and allowed free range following feeding. No additional treatments were administered throughout the experiment. The SCC remained below 2 × 10^5^ cells/mL for five consecutive days prior to the experiment measured by SCC-100 (ChemoMetec, Allerod, Denmark). Given immunological crosstalk between udder quarters [27], a paired design was used. The left hind quarters were designed as infection group, and the right hind quarters were designed as control group within the same cow to control individual variation.

After morning milking, the nipples were disinfected with iodophor. Then, the left hind quarters (*n* = 6) of each cow were injected with 5 mL saline containing 1 × 10^8^ CFU/mL of *K. pneumoniae* as the infection group, and the right hind quarters (*n* = 6) were infused with 5 mL sterile saline as the control group. After the injection, the udder was massaged to facilitate the distribution of *K. pneumoniae*. Milk samples (50 mL) and mammary acinar tissues were collected 24 h post-injection. During each milk sample collection, the first three milking samples were discarded to ensure aseptic conditions. After anesthetizing dairy cows with xylazine hydrochloride (0.1 mg/kg body weight, intramuscular injection), mammary acini tissues adjacent to lactiferous ducts were sampled using biopsy punches (BPP-40F, Kai Industries Co., Ltd, Japan).

Milk samples were used to detect the trace elements. The mammary acini tissues frozen in liquid nitrogen were used for RNA and protein extraction, and tissues fixed in 4% paraformaldehyde were used for hematoxylin-eosin (HE) staining, Prussian iron staining, ROS staining, and immunohistochemical staining.

### Detection of milk trace elements

The concentrations of trace elements in milk samples were determined using inductively coupled plasma mass spectrometry (PreMed 7000, EXPECLIN, Hangzhou, China). According to the instruction manual, 150 μL of the milk sample was diluted with 2.85 mL of double-distilled water, and the mixture was vortexed uniformly. The diluted milk sample was left to stand for 15 min before the analysis. Blank solutions were prepared by following the same procedure, except that no milk samples were added. Double-distilled water was used for cleaning glassware, diluting digests, and preparing standard solutions.

### Real-time quantitative PCR (RT-qPCR)

Total RNA was extracted from mammary glands or BMECs using the TransZol Up Kit (ET111-01-V2, TransGen, Beijing, China). NanoDrop-2000 (Thermo Fisher Scientific, Waltham, MA, USA) was used to determine RNA concentration and purity. Subsequently, the TransScript First-Step RT-PCR SuperMix (AQ601, TransGen, Beijing, China) was used to reverse-transcribe RNA into cDNA. PCR was performed using SuperMix (AQ132-11, TransGen, Beijing, China) and the CFX96 Touch Real-Time PCR Detection System (Bio-Rad, Hercules, USA). Each sample was assayed in triplicate and gene expression levels were calculated in relation to *ACTB* using the 2^−ΔΔCt^ method. The primer sequences are listed in Table 1.

**Table 1.** List of primers for real-time quantitative PCR.

Gene	Primer	Sequence (5′-3′)	Product size (bp)	Access number
*ACTB*	F	CATCACCATCGGCAATGAGC	156	NM173979.3
	R	AGCACCGTGTTGGCGTAGAG		
*IL-1β*	F	AGGTCCATACCTGACGGCTA	134	NM174093.1
	R	TTGGGTGTCTCAGGCATCTC		
*IL-6*	F	TGAAAGCAGCAAGGAGACACT	90	NM173923.2
	R	TGATTGAACCCAGATTGGAAGC		
*IL-8*	F	TTCCTCAGTAAAGATGCCAATG	86	NM173925.2
	R	TGACAACCCTACACCAGACCCA		
*NFE2L2*	F	AGCCTCAAAGCACCGTCC	132	NM173966.3
	R	CAAATCCATGTCCTGCTGGGA		
*TNF-α*	F	GGGCTTTACCTCATCTACTCACAG	85	NM1011678.2
	R	GATGGCAGACAGGATGTTGACC		
*xCT*	F	GCCTTGTCCTACGCTGAACT	135	XM24977578.2
	R	GGCTGCAGGGCGTATAATGA		
*GPX4*	F	AATCCCAAGACCCGTGCG	150	NM1346430.1
	R	CTCATTGCGAGGCCACATTG		

### HE staining

Mammary acini tissues were collected from each hind mammary gland were fixed in a 4% formaldehyde solution for 24 h. The fixed tissues were subsequently rinsed with water and dehydrated using 70%, 80%, 95%, and 100% ethanol. After xylene transparency, mammary tissues were embedded in paraffin and cut into 4 µm paraffin sections. Deparaffinized rehydrated mammary sections were stained using HE staining solution (G1120, SolarBio, Beijing, China). The stained mammary sections were observed using a microscope and photographed (Axio Vert. A1, Carl Zeiss, Jena, Germany). Pathological scoring was performed according to the following criteria. Grade 1: minimal – histological features (necrosis, neutrophils, lymphocytes) are rare or very scant within the examined 200× magnification field. Grade 2: mild – histological characteristics (necrosis, neutrophils, or lymphocytes) are consistently present in low quantities across the 200× magnification fields, with preserved normal tissue architecture. Grade 3: moderate – histopathological features (necrosis, neutrophils, or lymphocytes) are prominent and distinct in the examined area, with most glands or tissues displaying focal necrosis or inflammatory infiltrates (neutrophils or lymphocytes). Grade 4: severe – helming histopathological changes (necrosis, neutrophils, or lymphocytes) dominate the 200× magnification field, typically affecting all glands in the region and obscuring normal glandular structure.

### Prussian iron staining

The mammary sections were deparaffinized. Staining was performed according to the instructions (G1428, SolarBio, Beijing, China). In brief, the Perls solution was dropped onto the sections at a ratio of 1:1 and then incubated at 37°C for 20 min. The sections were then rinsed with water, and the incubation solution was dropped onto them at a ratio of 1:9. The sections were incubated at 37°C for 20 min and washed three times with PBS. The enhanced working solution was dropped onto the sections at a ratio of 1:1:18 and incubated at 37°C for 20 min. Subsequently, the sections were stained with redyeing solution for 5 min, soaked in distilled water, dehydrated with gradient ethanol, cleaned with xylene, and sealed with resin. Yellow-brown staining indicates a positive result. Pathological changes were observed under a microscope (Axio Vert. A1, Carl Zeiss, Jena, Germany).

### ROS staining

Dihydroethidium (DHE) was used to visualize ROS accumulation in the mammary tissue sections. Briefly, the sections were incubated for 1 h in a 10 μM DHE solution (HY-D0079, MedChemExpress, Shanghai, China) in dark at 37°C. The sections were then washed thrice with PBS. Subsequently, the mammary tissue sections were counterstained with DAPI. The stained mammary sections were mounted using neutral balata and inspected using an SP8 fluorescence microscope (Leica, Wetzlar, Germany).

### Immunohistochemistry

Mammary sections were cultured in 3% hydrogen peroxide for 30 min. Then The sections were immersed in sodium citrate buffer at 100°C for 5 min. Then, the mammary sections were blocked with bovine serum albumin (BSA) for 20 min at room temperature and incubated overnight at 4°C with the primary antibody of anti-4-HNE antibody at 1:250 dilution overnight. The sections were then incubated with secondary antibodies (SA1020, Boster Biological Technology, Wuhan, China) for 30 min. Immunoreactivity was visualized using a 3, 3’-diaminobenzidine solution. Mammary sections were counterstained with hematoxylin and evaluated under a microscope (Axio Vert. A1, Carl Zeiss, Jena, Germany).

### BMECs culture and treatment

BMECs were isolated from Holstein cows diagnosed as free of mastitis and mycoplasma contamination by professional veterinarians. Mammary acini tissues were collected aseptically and washed with PBS containing 300 IU penicillin/streptomycin to remove milk and blood. The mammary acini tissues were then transported to the laboratory for further processing, as previously described [28]. Immunohistochemical staining for cytokeratin 18 was performed to ensure the purity of BMECs (Additional file 1). The BMECs were grown, passaged, and frozen for subsequent experiments. In this study, third-to sixth-generation BMECs were used. BMECs were challenged with *K. pneumoniae* at a multiplicity of infection (MOI) of 10:1 for various time points (0, 2, 4, 6, and 8 h). Furthermore, to identify ferroptosis function during infection, BMECs were cultured with 10 μM Fer-1 and/or *K. pneumoniae* at an MOI of 10:1 for 6 h, followed by samples collection and analysis in subsequent experiments.

### Western blot analysis

Mammary acini tissues were lysed in radioimmunoprecipitation assay (RIPA) lysis buffer (C1053, Applygen Technologies Inc, Beijing, China) for 30 min on ice. BMECs were lysed in RIPA lysis buffer for 15 min on ice. Subsequently, the lysate was centrifuged at 1.2 × 10^4^ rpm for 10 min, and the supernatant was used for western blotting. Proteins and molecular marker (DM131, TransGen, Beijing, China) were dissolved in SDS-PAGE loading buffer (P1040, SolarBio, Beijing, China), separated via electrophoresis. The proteins were then transferred onto PVDF membranes. Membranes were blocked with 5% skim milk and washed with TBST. The membranes were incubated with different antibodies, including anti-PTGS2, anti-Nrf2, anti-xCT, and anti-GPX4 antibodies overnight at 4°C. The membranes were then washed with TBST. After incubation with goat anti-rabbit IgG secondary antibody (#31460, 1:10000 dilution, Thermo Fisher Scientific, Waltham, USA), the membrane was washed with TBST, and the expression levels of proteins were detected using a chemiluminescent assay kit (1810202, Clinx, Shanghai, China).

### Assessment of LDH, GSH and GSH-Px

The lactate dehydrogenase (LDH) was detected using an assay kit in mammary acini or BMECs culture supernatants (A020-2–2, Nanjing Jiancheng Bioengineering Institute, Nanjing, China). In short, the mammary tissue lysates or BMECs culture supernatants reacted with coenzyme I, 2, 4 dinitrophenylhydrazine was added to continue to react, and then sodium hydroxide solution was added to react. The LDH activity in the samples was calculated based on the optical density at 440 nm (Epoch 2, BioTek Instruments, Inc., Winooski, USA). Additionally, GSH content in mammary acini or BMECs was detected using a kit (S0053, Beyotime Biotechnology, Shanghai, China). Briefly, lysates were prepared and GSH removal reagent was added, followed by total glutathione working solution and NADPH. The optical density at 412 nm represents the GSSG. In parallel tests, the GSH removal reagent was omitted, and the optical density at 412 nm represented the total GSH + GSSG content (Epoch 2, BioTek Instruments, Inc., Winooski, USA). The GSH content was calculated by subtracting GSSG from GSH + GSSG. The activity of glutathione peroxidase (GSH-Px) in mammary acini or BMECs was detected using a kit (A005-1–2, Nanjing Jiancheng Bioengineering Institute, Nanjing, China). Briefly, H_2_O_2_ and GSH were added to the samples, and GSH-Px activity was measured using the dinitrothiocyano benzene method. The GSH-Px activity in the samples was calculated by measuring the optical density at 412 nm (Epoch 2, BioTek Instruments, Inc., Winooski, USA).

### Immunofluorescence analysis

Mammary sections were deparaffinized and antigen retrieved, blocked with 5% BSA, followed by incubation with the anti-Nrf2 antibody at 4°C overnight. The sections were then incubated with goat anti-rabbit secondary antibody (#A32731, 1:200 dilution, Thermo Fisher Scientific, Waltham, USA) for 1.5 h at 37°C and counterstained with DAPI for 15 min. BMECs were cultured on coverslips. BMECs were cultured with Fer-1 and/or *K. pneumoniae* for 6 h. BMECs were fixed in methanol and blocked using 5% BSA for 15 min at room temperature. The cell membranes were permeabilized by treatment with 0.3% Triton X-100 for 15 min. Then the coverslips were washed with PBS, and the BMECs were incubated with the anti-Nrf2 antibody at 4°C overnight. The subsequent steps were performed under light-protected conditions. The BMECs were then incubated with a goat anti-rabbit secondary antibody (#A32731, 1:200 dilution, Thermo Fisher Scientific, Waltham, USA) for 1.5 h at 37°C and counterstained with DAPI for 15 min. Finally, the distribution of Nrf2 nuclear translocation was observed by SP8 laser confocal microscopy (Leica, Wetzlar, Germany). The plot profile of ImageJ was used to generate spatial intensity curves for Nrf2 (green) and nuclei (blue) fluorescence in epithelial cells of mammary glands.

### Assessment of intracellular Fe^2+^ and lipid ROS

The 1 µM FerroOrange (F347, Dojindo Molecular Technologies, Inc., Kumamoto, Japan) was used to assess the concentration of intracellular Fe^2+^. Iron was chelated with 5 μM deferoxamine mesylate (HY-B1625, MedChemExpress, Shanghai, China) as a negative control. Cells were assessed using an SP8 confocal laser microscope (Leica, Wetzlar, Germany). To evaluate lipid ROS levels, BMECs were treated with 5 μM Liperfluo (L248, Dojindo, Kumamoto, Japan). The median FITC fluorescence intensity was measured by flow cytometry (CytoFLEX S, Becton Coulter, Inc., Brea, CA, USA) to represent the level of lipid ROS in the cell membrane. For each sample, 1 × 10^4^ cells were collected.

### Assessment of mitochondrial superoxide

To capture the changes in mitochondrial superoxide during *K. pneumoniae* invasion of BMECs, 5 µM MitoSOX^TM^ mitochondrial superoxide indicator were incubated with cells (M36228, Thermo Fisher Scientific, Waltham, USA) for 50 min at 37°C. The BMECs were then washed with phosphate-buffered saline (PBS). Nuclei were stained with Hoechst (B2261, Sigma-Aldrich, St. Louis, USA). Laser confocal microscopy (SP8, Leica, Wetzlar, Germany) was used to observe and capture mitochondrial superoxide, while ImageJ was used for fluorescence intensity analysis.

### Transmission electron microscopy observation

The BMECs were harvested and fixed with 2.5% glutaraldehyde. The BMECs were then fixed with 1% osmium tetroxide for 2 h. Gradient dehydration was then performed using ethanol. The BMECs were embedded in the resin and cured to produce solid blocks, which were then sectioned into 65 nm slices using a microtome. Sections were stained with a solution containing 1% uranyl acetate and 0.1% lead citrate. Stained sections were observed under a transmission electron microscope (HT7800, HITACHI, Tokyo, Japan).

### Cell viability determination

To evaluate the effect of Fer-1 on the cell viability, cells were seeded in 96-well plates at a density of 1 × 10^4^ cells/well. Briefly, cells were treated with 0.1% dimethyl sulfoxide (DMSO) or different concentrations of Fer-1 (1, 5, 10, 20, 50, 100 μM) for 24 h. Then, the BMECs were incubated with 10 μL CCK-8 solution (A311-01, Vazyme, Nanjing, China) at 37°C for 2 h. Cell viability was calculated by measuring optical density at 450 nm (Epoch 2, BioTek Instruments, Inc., Winooski, USA).

### Measurement of adherent bacterial load

To evaluate the effect of ferroptosis inhibition on the bacterial load of BMECs, the cells were seeded in 24-well cell plates at a concentration of 2 × 10^4^ cells/well. The BMECs were cultured with Fer-1 and/or *K. pneumoniae* for 6 h. Then, the cells were washed six times with PBS to remove the non-adherent *K. pneumoniae*. 0.3% Triton X-100 was used for 15 min to lyse BMECs, which were serially diluted and cultured on LB agar medium. The number of *K. pneumoniae* colonies was counted and recorded.

### Statistical analysis

Statistical analysis was performed using GraphPad Prism 9.0 (Graph Pad Software, La Jolla, CA, USA). The unpaired t-test was used to compare the two groups. One-way analysis of variance (ANOVA) was performed for multiple comparisons with Bonferroni correction for data meeting the homogeneity of variance. Data were expressed as the mean ± SD. *p* < 0.05 was considered statistically significant and *p* < 0.01 markedly significant.

## Results

### *K.* *pneumoniae* challenge caused ferroptosis in bovine mammary glands

**Figure 1.**
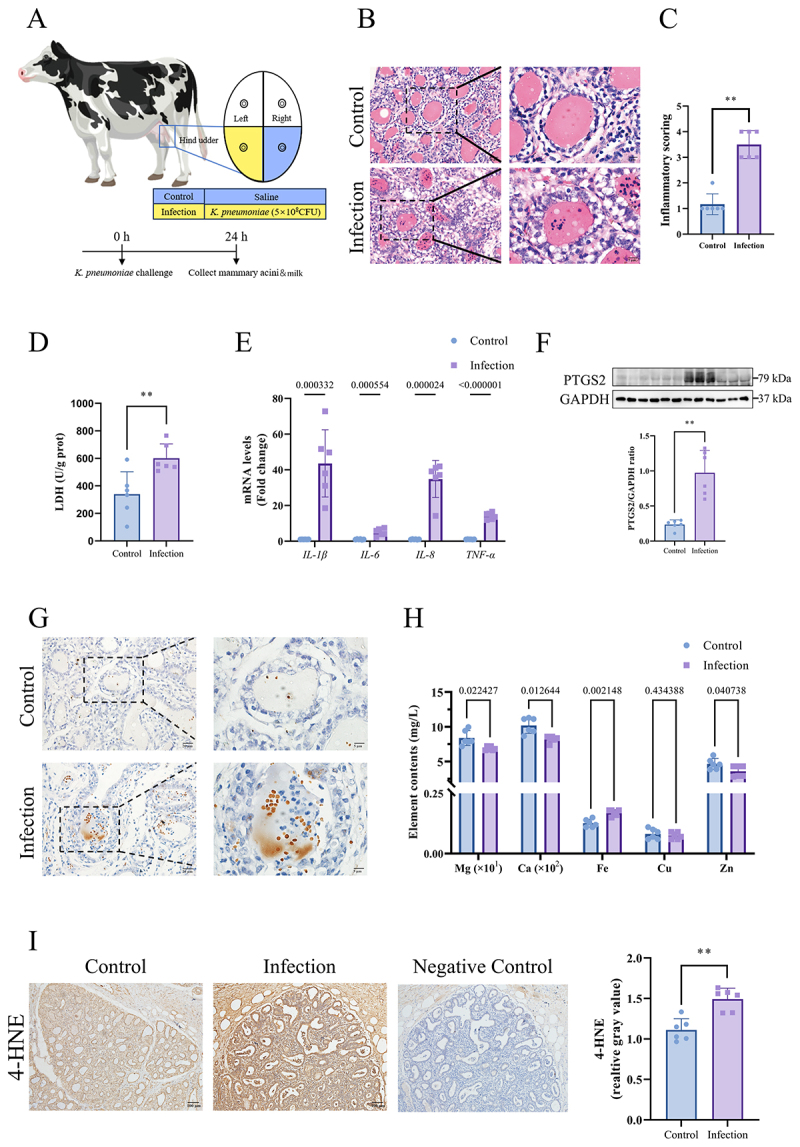
*K. pneumoniae* challenge caused ferroptosis in mammary acini. (A) The left hind quarters of dairy cows were injected with 5 mL of saline containing 1 × 10^8^ CFU/mL of *K. pneumoniae* as the infection group. The right hind quarters were injected with 5 mL saline as the control group. (B) Histological evaluation of mammary tissue was conducted using he staining at magnifications of 40× and 1,00×. (C) HE-based inflammatory scoring. (D) LDH activity in mammary tissues. (E) mRNA expression levels of inflammatory factors *IL-1β*, *IL-6*, *IL-8* and *TNF-α* were detected by RT-qPCR. (F) Western bolt was used to detect PTGS2 protein levels in control group and infected group. (H) Observation of ferric iron (Fe^3+^) concentration variation in the mammary glands using Prussian iron staining. (H) The contents of magnesium (Mg), calcium (Ca), ferrum (Fe), copper (Cu) and zinc (zu) in milk were detected by inductively coupled plasma mass spectrometry. (I) The level of 4-HNE in control group and infection group was detected by immunohistochemistry at magnifications of 40 × . **p < 0.01 versus the control group. Data were expressed as mean ± SD. Each point in the bar graphs represents a biological repetition.

Bovine mammary tissues were infected with *K. pneumoniae* to evaluate ferroptotic signatures (Figure 1(A)). The findings revealed histological alterations within the infected mammary acini, characterized by alveolus thickening, edema, hemorrhage, and neutrophil infiltration (Figure 1(B)). Inflammatory scoring revealed severe inflammation (grade 3–4, *p* < 0.01, Figure 1(C)) in infected mammary acini, with predominant neutrophilic infiltrates (multilobed nuclei, granular cytoplasm) in periglandular zones. Control mammary acini scored grade 1–2 (minimal infiltrates, Figure 1(C)). Following *K. pneumoniae* challenge, significant elevations in LDH activity (*p* < 0.01, Figure 1(D)) and mRNA expression of *IL-1β*, *IL-6*, *IL-8*, and *TNF-α* (*IL-1β, p* = 0.000332, *IL-6, p* = 0.000554, *IL-8, p* = 0.000024, and *TNF-α, p* < 0.000001, Figure 1(E)) were observed in comparison to the control group. The expression of the PTGS2 protein, a pivotal biomarker for ferroptosis [29], was significantly increased in the infection group (*p* < 0.01, Figure 1(F)). The infection group exhibited significantly elevated iron concentrations in both mammary acini (Figure 1(G)) and milk samples (*p* = 0.002148, Figure 1(H)), in stark contrast to those in the control group. In addition, the concentrations of magnesium (Mg), calcium (Ca) and zinc (Zn) in milk samples from the infected group were lower than those of the control group, whereas the copper (Cu) content showed no significant change (Mg, *p* = 0.022427, Ca, *p* = 0.012644, Zn = 0.040738, and Cu = 0.434388, Figure 1(H)). Furthermore, 4-HNE, a key indicator of lipid peroxidation, was notably increased in the infection group (*p* < 0.01, Figure 1(I)).

### The Nrf2/xCT/GPX4 pathway was inhibited in mammary tissues with ferroptosis increase induced by *K.* *pneumoniae* invasion

**Figure 2.**
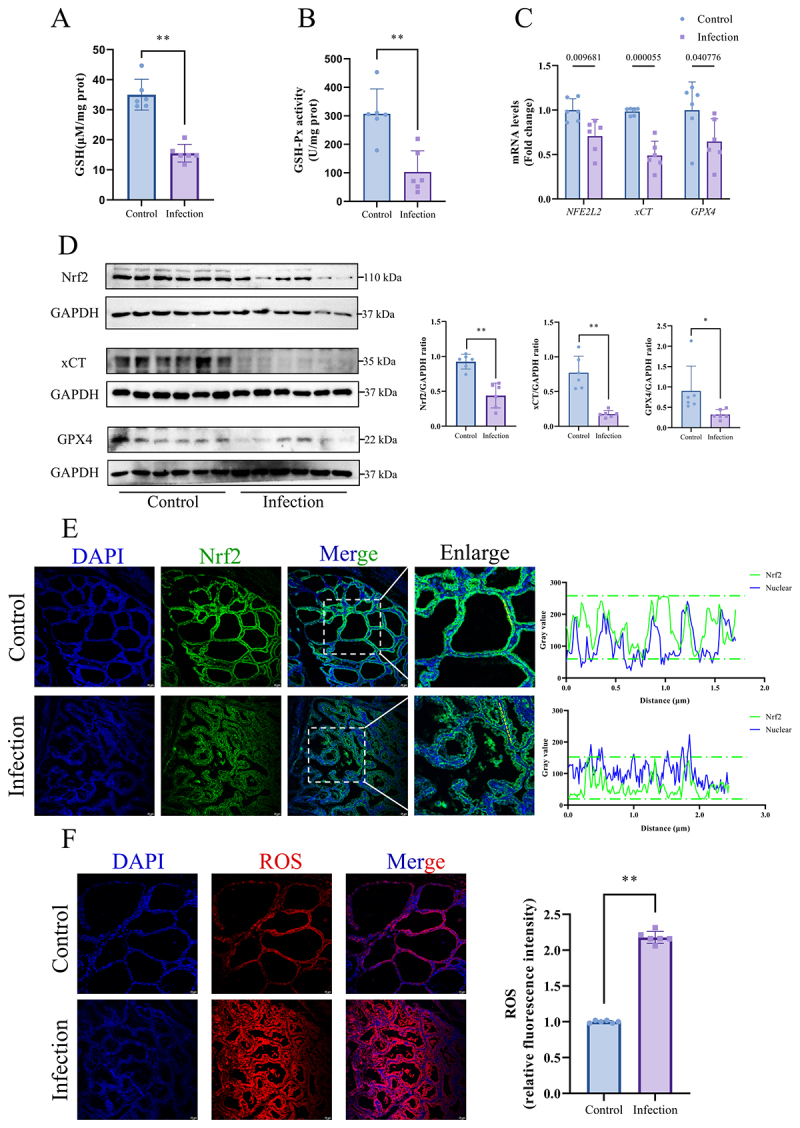
*K. pneumoniae* inhibited Nrf2/xCT/GPX4 signaling pathway in mammary gland of dairy cows. (A) The content of GSH and the (B) Activity of GSH-Px in mammary tissues. (C) The mRNA levels of *NFR2L2*, *xCT* and *GPX4* in mammary tissues of control group and infection group were detected by RT-qPCR. (D) Nrf2, xCT and GPX4 protein levels were detected and quantified by Western blot. (E) immunofluorescence observations the distribution of Nrf2 in the control group and infection group. Bar = 20 μm. (F) Dihydroethidium staining was utilized to visualize ROS accumulation in mammary tissue sections, and carry-on quantitative analysis. Bar = 20 μm. *p < 0.05; **p < 0.01 versus the control group. Data were expressed as mean ± SD. Each point in the bar graphs represents a biological repetition.

Quantitative assessment revealed that both GSH (*p* < 0.01, Figure 2(A)) and GSH-Px activity (*p* < 0.01, Figure 2(B)) in mammary tissues were sharply reduced in the infection group compared to those in the control group. The expression profiles of key Nrf2 pathway components, including Nrf2, xCT, and GPX4, were decreased at both the transcript (*NFE2L2, p* = 0.009681, *xCT, p* = 0.000055, and *GPX4, p* = 0.040776, Figure 2(C)) and protein levels (*p* < 0.05 or *p* < 0.01, Figure 2(D)) in the infection group compared to the control group. Immunofluorescence analysis revealed differential subcellular localization of Nrf2. In the control group, Nrf2 and DAPI intensity curves exhibited peak alignment (intensity maxima overlapping at nuclear coordinates), confirming nuclear colocalization. Conversely, the infection group showed that Nrf2 intensity peaks were displaced from DAPI maxima, with curves showing minimal overlap (Figure 2(E)). In parallel, we observed an elevation in ROS levels, as evidenced by the heightened red fluorescence intensity in the infection group (*p* < 0.01, Figure 2(F)).

### *K.* *pneumoniae* induced ferroptosis in BMECs

**Figure 3.**
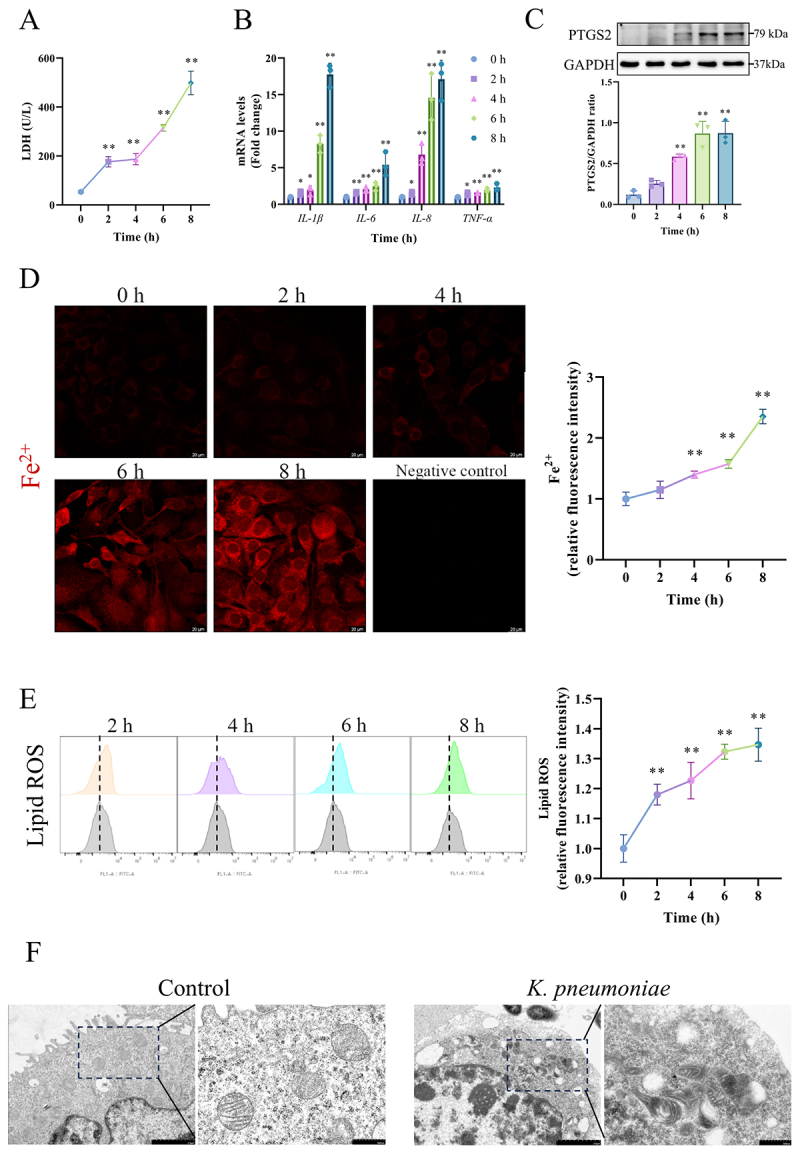
*K. pneumoniae* infection induced ferroptosis in BMECs. (A) The LDH activity in the culture medium supernatant at different time points after *K. pneumoniae* invasion. (B) The mRNA levels of *IL-1β*, *IL-6*, *IL-8* and *TNF-α* were detected by RT-qPCR. (C) Western immunoblotting was used to assess PTGS2 levels over time. (D) FerroOrange was used to detect intracellular Fe^2+^ level. The mean fluorescence intensity represents the intracellular Fe^2+^ level observed using laser confocal microscopy. Negative control used 5 μM deferoxamine mesylate to chelate iron. Bar = 20 μm. (E) Liperfluo was used to detect the BMECs’ lipid ROS levels by flow cytometry. The median FITC fluorescence intensity represents the lipid ROS. (F) The mitochondrial morphology was observed by transmission electron microscope. Bar = 2 μm or bar = 500 nm. The BMECs were challenged with *K. pneumoniae* at a multiplicity of infection of 10:1 for various time points (0, 2, 4, 6, and 8 h). *p < 0.05, **p < 0.01 versus 0 h. Data were expressed as mean ± SD. Each point in the bar graphs represents a biological repetition.

An in vitro *K. pneumoniae* infection model was constructed using BMECs. The LDH released from BMECs markedly increased, peaking at 8 h post-*K. pneumoniae* infection (*p* < 0.01, Figure 3(A)). The mRNA expression of *IL-1β*, *IL-6*, *IL-8*, *TNF-α* (*p* < 0.05 or *p* < 0.01, Figure 3(B)) and the protein abundance of PTGS2 (*p* < 0.01, Figure 3(C)) increased in BMECs over the course of *K. pneumoniae* infection. Notably, intracellular Fe^2+^ concentrations increased, exhibiting a 1.399-fold increase at 4 h, 1.574-fold at 6 h, and peaked at 2.352-fold at 8 h post-infection (*p* < 0.01, Figure 3(D)). The generation of lipid ROS was augmented at 2 h post-infection and progressively intensified with the continuation of *K. pneumoniae* infection (*p* < 0.01, Figure 3(E)). Ultrastructural analysis showed that *K. pneumoniae* adhered to the exterior of BMECs, accompanied by increased mitochondrial membrane density and the disappearance or blurring of mitochondrial cristae compared with the control group (Figure 3(F)).

### *K. pneumoniae*-induced ferroptosis in BMECs was associated with the inhibition of Nrf2/xCT/GPX4 pathway

**Figure 4.**
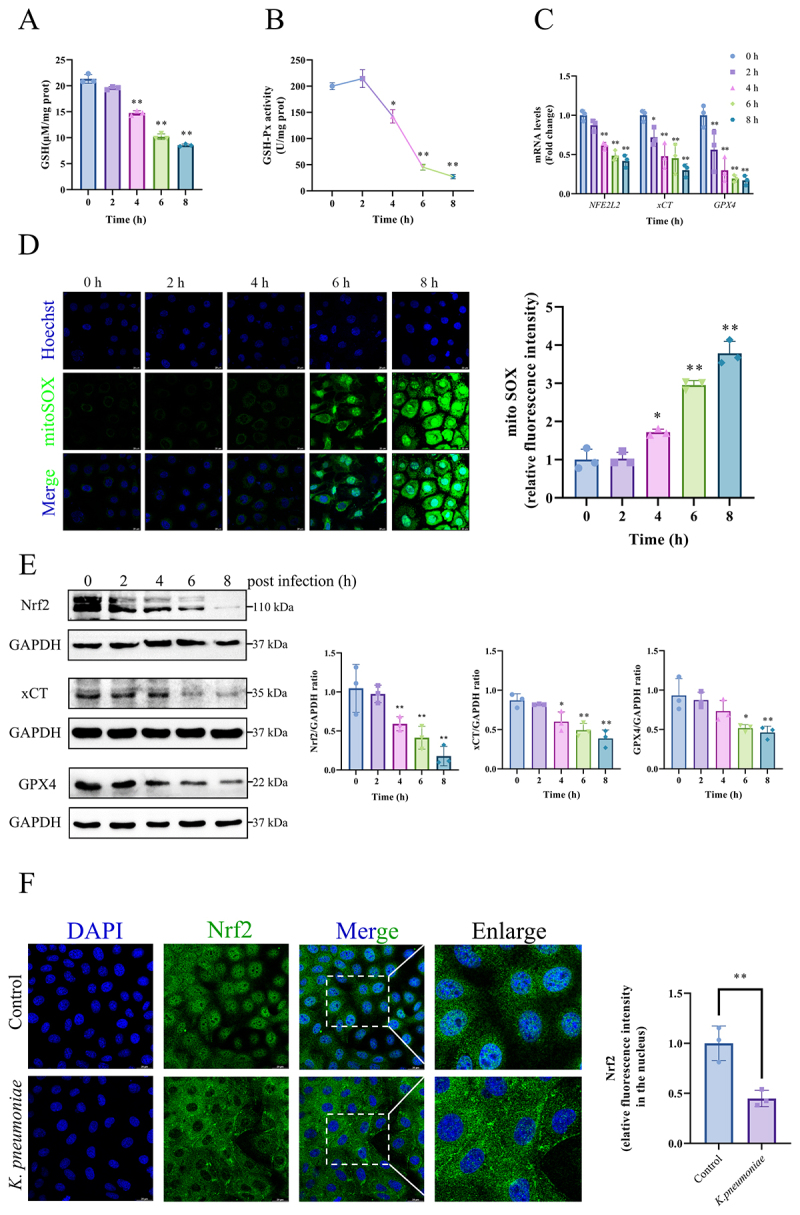
*K. pneumoniae* infection led to inhibition of the Nrf2/xCT/GPX4 signaling pathway in BMECs. (A) The content of GSH and the (B) Activity of GSH-Px in BMECs. (C) The mRNA levels of *NFR2L2*, *xCT* and *GPX4* were detected by RT-qPCR. (D) Mito sox probe was used to measure mitochondrial superoxide levels in BMECs. The mean fluorescence intensity represents the levels of mitochondrial superoxide observed by laser confocal microscopy. Bar = 20 μm. (E) Nrf2, xCT and GPX4 protein levels were detected and quantified by Western blot. (F) The nuclear translocation of Nrf2 was observed by immunofluorescence. Bar = 20 μm. The BMECs were challenged with *K. pneumoniae* at a multiplicity of infection of 10:1 for various time points (0, 2, 4, 6, and 8 h). *p < 0.05, **p < 0.01 versus 0 h. Data were expressed as mean ± SD. Each point in the bar graphs represents a biological repetition.

With the continuation of *K. pneumoniae* infection, the activities of GSH (*p* < 0.01, Figure 4(A)) and GSH-Px in BMECs were inhibited (*p* < 0.05 or *p* < 0.01, Figure 4(B)). The mRNA expression of *NFE2L2*, *xCT*, and *GPX4* was downregulated in response to *K. pneumoniae* invasion (*p* < 0.05 or *p* < 0.01, respectively, Figure 4(C)). Mitochondrial superoxide accumulation surged 4 h post-infection (*p* < 0.05, Figure 4(D)) and continued to increase with the duration of infection (*p* < 0.01, Figure 4(D)). Western blot analysis revealed suppression of Nrf2/xCT/GPX4 pathway protein expression (*p* < 0.05 or *p* < 0.01, Figure 4(E)) upon *K. pneumoniae* infection. In *K. pneumoniae*-infected cells, immunofluorescence staining demonstrated that Nrf2 localization shifted predominantly to the cytoplasm, with minimal co-localization with the nucleus, suggesting impaired nuclear translocation. Moreover, the intensity of green fluorescence in the nucleus was reduced. Conversely, in the control group, immunofluorescence staining demonstrated that Nrf2 (green fluorescence) was present in both the nucleus and cytoplasm, with clear colocalization indicating nuclear translocation. (*p* < 0.01, Figure 4(F)).

### Fer-1 mitigated *K. pneumoniae*-induced BMECs damage by inhibiting ferroptosis

**Figure 5.**
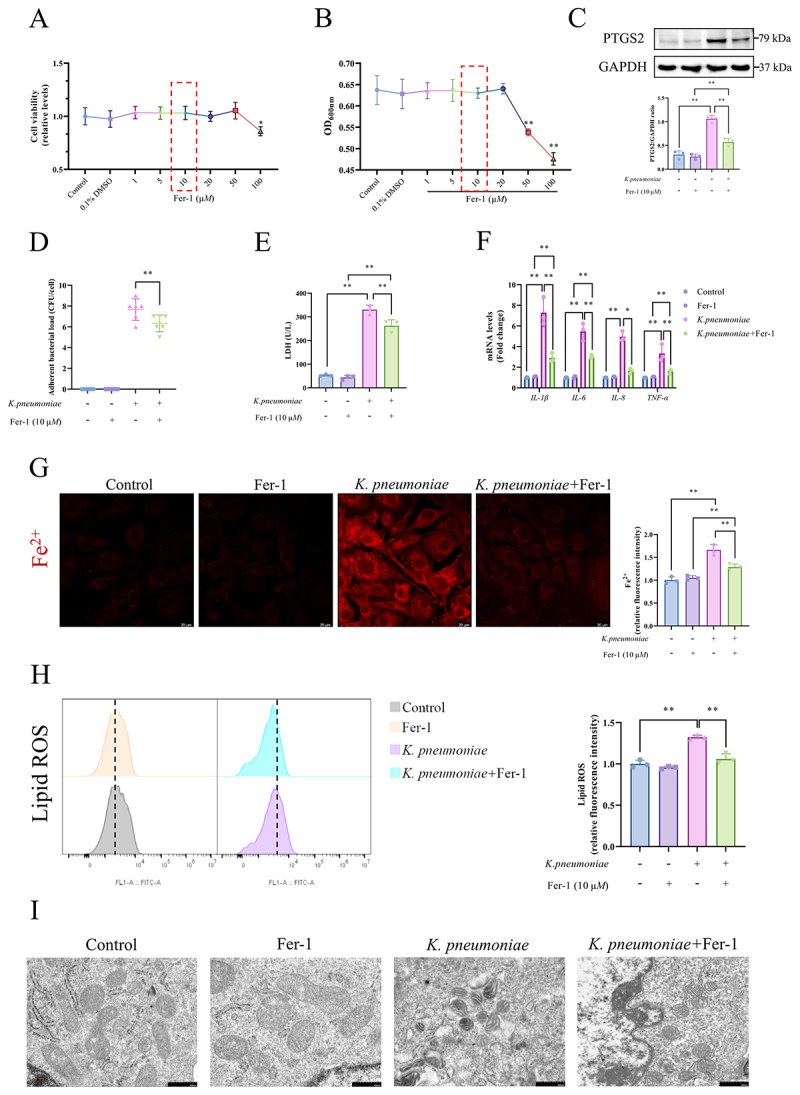
Fer-1 alleviated *K. pneumoniae*-induced BMECs injury by inhibiting ferroptosis. (A) The effect of Fer-1 on BMECs viability was determined by the CCK-8 assay, and (B) The effect of Fer-1 on *K. pneumoniae* growth has been assessed by measuring optical density at 600 nm. (C) Western blot was used to assess PTGS2 levels. (D) The effect of Fer-1 on adherent *K. pneumoniae* load in BMECs was determined by plate dilution coating method. (E) The LDH concentration in the culture medium supernatant. (F) mRNA expression levels of inflammatory factors *IL-1β*, *IL-6*, *IL-8* and *TNF-α* were detected by RT-qPCR. (G) FerroOrange was used to detect intracellular Fe^2+^ levels. The mean fluorescence intensity represents the intracellular Fe^2+^ levels observed using laser confocal microscopy. Bar = 20 μm. (H) Liperfluo was used to detect the BMECs lipid ROS levels by flow cytometry. The median FITC fluorescence intensity represents the lipid ROS. (I) The mitochondrial morphology was observed by transmission electron microscope. Bar = 500 nm. BMECs was treated with 10 μM Fer1 and *K. pneumoniae* alone or in combination for 6 h with a multiplicity of infection of 10:1. *p < 0.05, **p < 0.01. Data were expressed as mean ± SD. Each point in the bar graphs represents a biological repetition.

To verify the effect of ferroptosis inhibition on *K. pneumoniae* infection of BMECs, the ferroptosis inhibitor Fer-1 was used to co-treat BMECs with *K. pneumoniae*. Fer-1 (10 μM) exhibited neutrality toward BMECs cellular activity (Figure 5(A)) and did not impede the proliferation of *K. pneumoniae* (Figure 5(B)). Therefore, Fer-1 at a concentration of 10 μM was used in the subsequent experiments. Contrasted with the *K. pneumoniae*-infected group, concurrent administration of Fer-1 downregulated PTGS2 protein expression (*p* < 0.01, Figure 5(C)), mitigated adherent bacterial load (*p* < 0.01, Figure 5(D)), and curtailed LDH release (*p* < 0.01, Figure 5(E)) in BMECs. Additionally, Fer-1 effectively suppressed the transcription of the inflammatory cytokines *IL-1β*, *IL-6*, *IL-8*, and *TNF-α* (*p* < 0.05 or *p* < 0.01, Figure 5(F)) induced by *K. pneumoniae*. Furthermore, when ferroptosis was inhibited, Fe^2+^ and lipid ROS accumulation was mitigated. (*p* < 0.01, Figure 5(G,H)). Ultrastructural analysis revealed that, in stark contrast to *K. pneumoniae* infection group, the group subjected to both Fer-1 and *K. pneumoniae* displayed restoration of mitochondrial double membrane integrity and crest structure (Figure 5(I)).

### Fer-1 protected BMECs from *K.* *pneumoniae* damage via activation of the Nrf2/xCT/GPX4 pathway

**Figure 6.**
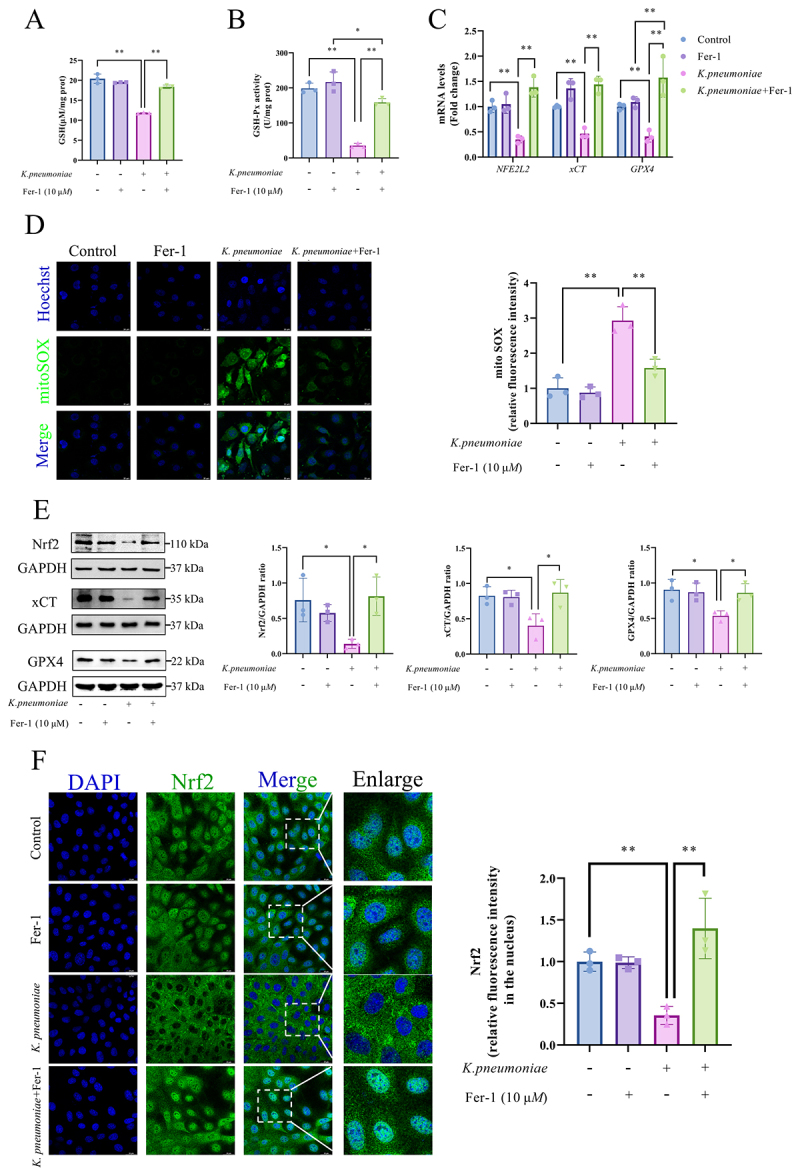
Fer-1 protected BMECs from *K. pneumoniae* damage by activating the Nrf2/xCT/GPX4 signaling pathway. (A) The content of GSH and (B) The activity of GSH-Px in BMECs. (C) The levels of *NFR2L2*, *xCT* and *GPX4* were detected by RT-qPCR. (D) Mito sox probe was used to measure mitochondrial superoxide levels in BMECs. The mean fluorescence intensity represents the levels of mitochondrial superoxide observed by laser confocal microscopy. Bar = 20 μm. (E) Nrf2, xCT and GPX4 protein levels were detected and quantified by Western blot. (F) The nuclear translocation of Nrf2 was observed by immunofluorescence. Bar = 20 μm. BMECs was treated with 10 μM Fer-1 and *K. pneumoniae* alone or in combination for 6 h with a multiplicity of infection of 10:1. *p < 0.05, **p < 0.01. Data were expressed as mean ± SD. Each point in the bar graphs represents a biological repetition.

Compared with the *K. pneumoniae*-infected group, GSH (*p* < 0.01, Figure 6(A)) and GSH-Px (*p* < 0.01, Figure 6(B)) activities were significantly increased in BMECs co-treated with Fer-1 and *K. pneumoniae* group. Furthermore, Fer-1 treatment enhanced the mRNA transcription of *NFE2LE*, *xCT*, and *GPX4* (*p* < 0.01, Figure 6(C)) while effectively reducing mitochondrial superoxide accumulation (*p* < 0.01, Figure 6(D)) induced by *K. pneumoniae* infection. Western blotting analysis confirmed an increase in the protein levels of Nrf2, xCT, and GPX4 in the co-treated Fer-1 and *K. pneumoniae* group, in contrast to the *K. pneumoniae*-infected group (*p* < 0.05, Figure 6(E)). Immunofluorescence staining further corroborated these findings, showing a clear colocalization of Nrf2 (green fluorescence) with the nucleus (blue fluorescence) in the co-treated Fer-1 and *K. pneumoniae* group, as compared to the *K. pneumoniae*-infected group (*p* < 0.01, Figure 6(F)).

## Discussion

This study provides the first evidence that *K. pneumoniae* infection induces ferroptosis in both mammary tissues and BMECs via inhibition of the Nrf2/xCT/GPX4 pathway, while inhibition of ferroptosis effectively alleviates this process. These findings provide a rationale for the development of intervention strategies for dairy mastitis targeting ferroptosis.

Ferroptosis is a key contributor to bacterial infections [3,30,31]. However, the lack of experimental validation suggests that the pathogenic mechanism of *K. pneumoniae* is associated with ferroptosis. In this study, inflammatory cells and red blood cells were observed in mammary acini infected with *K. pneumoniae*, resulting in iron overload and lipid ROS accumulation. *K. pneumoniae* utilizes multiple siderophores (e.g. aerobactin encoded by *iutA*, yersiniabactin encoded by *ybtS*) to compete with host iron-binding proteins, disrupting systemic iron homeostasis [32]. Lipopolysaccharide-induced mastitis disrupts the blood-milk barrier by altering claudin composition (e.g. claudin-3 phosphorylation and claudin-4/7 recruitment) in mammary alveolar tight junctions, facilitating the influx of neutrophils and red blood cells into mammary acini [33]. Infiltration of neutrophils exacerbates oxidative stress through two mechanisms: (1) ROS overproduction via NADPH oxidase activation and (2) extracellular trap formation containing DNA-histone complexes and proteolytic enzymes (e.g. myeloperoxidase), which further damages the mammary epithelium [34,35]. Ultimately, these factors may culminate in the accumulation of iron and lipid peroxides within the mammary gland acini, thereby driving the progression of ferroptosis in cow mastitis.

*K. pneumoniae* infection triggered transcriptional upregulation of *IL-1β*, *IL-6*, *IL-8*, and *TNF-α* within 2 h post-infection, preceding significant changes in ferroptosis markers. IL-1β leads to the overload of Fe^2+^ and inhibits the expression of GPX4 and xCT in chondrocytes [36]. Additionally, 50 ng/mL of IL-6 inhibits the expression of GPX4 and thereby promote ferroptosis in human chondrocytes [37]. Consistent with our results, inflammatory cytokines were upregulated as the infection duration prolonged. Subsequently, the protein levels of xCT and GPX4 began to significantly decrease 4 h to 8 h post-infection, and the content of GSH also decreased accordingly. The inactivation of the antioxidant system eventually leads to the accumulation of lipid peroxides in BMECs, further promoting the expression of inflammatory factors. After inhibiting the activity of GPX4 in the hippocampus of rats, the expression of IL-1β and IL-18 was promoted [38]. In this study, after the cellular antioxidant system was inactivated (6 to 8 h post-infection), the inflammatory cytokines mRNA *IL-1β*, *IL-6*, *IL-8*, and *TNF-α* further increased and reached the maximum value 8 h post-infection. Thus, there is a bidirectional amplification loop during *K. pneumoniae* infection of BMECs wherein inflammation initiates ferroptosis, and ferroptosis subsequently exacerbates inflammation.

*K. pneumoniae* infection significantly inhibited the mRNA and protein expressions of Nrf2, xCT, and GPX4. Downregulation of xCT limits cystine uptake, thereby depleting intracellular GSH and impairing the reduction of lipid peroxides [39]. Concomitant suppression of GPX4 abolishes its capacity to detoxify lipid hydroperoxides, thereby accelerating membrane peroxidation and mitochondrial damage [40]. *K. pneumoniae* infection decreased GSH activity and also caused a decrease in GSH-Px activity. The collapse of mitochondrial cristae disrupts electron transport chain complexes, leading to electron leakage and excessive superoxide generation [41]. The results showed shrunken mitochondria with disintegrated cristae, increased membrane density, and mitochondrial SOX accumulation in *K. pneumoniae* infected BMECs. The combined disruption of xCT and GPX4 functions creates a self-amplifying oxidative stress cycle that drives ferroptotic death in BMECs.

Fer-1 has shown therapeutic effects in sepsis [20,42]. However, Fer-1 is currently not used for the treatment of bovine mastitis. 10 μM Fer-1alleviates *Staphylococcus aureus*-induced HC11 cells ferroptosis by reducing the inflammatory response, improving lipid peroxidation and restoring the antioxidant capacity of cells [43]. Administration of 10 μM Fer-1 attenuates lipid peroxidation in *Mycobacterium bovis*-infected RAW264.7 macrophage cells, reduces bacterial burden, and restores GPX4 protein expression [44]. Additionally, 10 μM Fer-1 ameliorated Toxoplasma gondii infection-induced lipid peroxidation and downregulated LDH production in macrophage cells [45]. Building on established efficacy of 10 μM Fer-1 in mitigating ferroptosis across infection models, 10 μM Fer-1 was employed to inhibit *K. pneumoniae*-induced ferroptosis in BMECs.

Fer-1 treatment suppressed the mRNA overexpression of inflammatory cytokines (*IL-1β*, *IL-6*, *IL-8*, *TNF-α*) in *K. pneumoniae*-infected BMECs, lowered adherent bacterial loads, diminished LDH leakage, and restored mitochondrial bilayer membrane integrity. Consistent with these findings, Fer-1 attenuated sepsis-induced upregulation of TNF-α, IL-1β, and IL-6 levels and improved cardiac function in a mice model of sepsis [30,46]. Interestingly, Fer-1 treatment significantly alleviated intracellular Fe^2+^ accumulation induced by *K. pneumoniae*. This might be related to the formation of complexes between Fer-1 and Fe^2+^, decreasing labile iron in cells [47]. In addition, Fer-1 inhibits ferroptosis in neuronal cells by upregulating the Nrf2/heme oxygenase-1 signaling pathway in a mouse model induced by cecal ligation and puncture [48]. Fer-1 alleviated the inhibition of Nrf2 nuclear translocation, increased xCT and GPX4 gene and protein expression, and enhanced GSH and GSH-Px activity in *K. pneumoniae* infected BMECs. Therefore, Fer-1 not only ameliorates oxidative stress and inflammatory cascades in BMECs triggered by *K. pneumoniae* but also inhibits ferroptosis through the activation of the Nrf2/xCT/GPX4 signaling cascade. However, the short half-life of Fer-1 *in vivo* and its difficulty in penetrating the blood-milk barrier limit its clinical application [49]. Moreover, the potential effects of Fer-1 on the lactation function of dairy cows are also unknown. Therefore, the next step can be to prepare an antibacterial hydrogel containing Fer-1 in combination with antibiotics and evaluate its discarding period after application in dairy cows

## Conclusion

**Figure 7.**
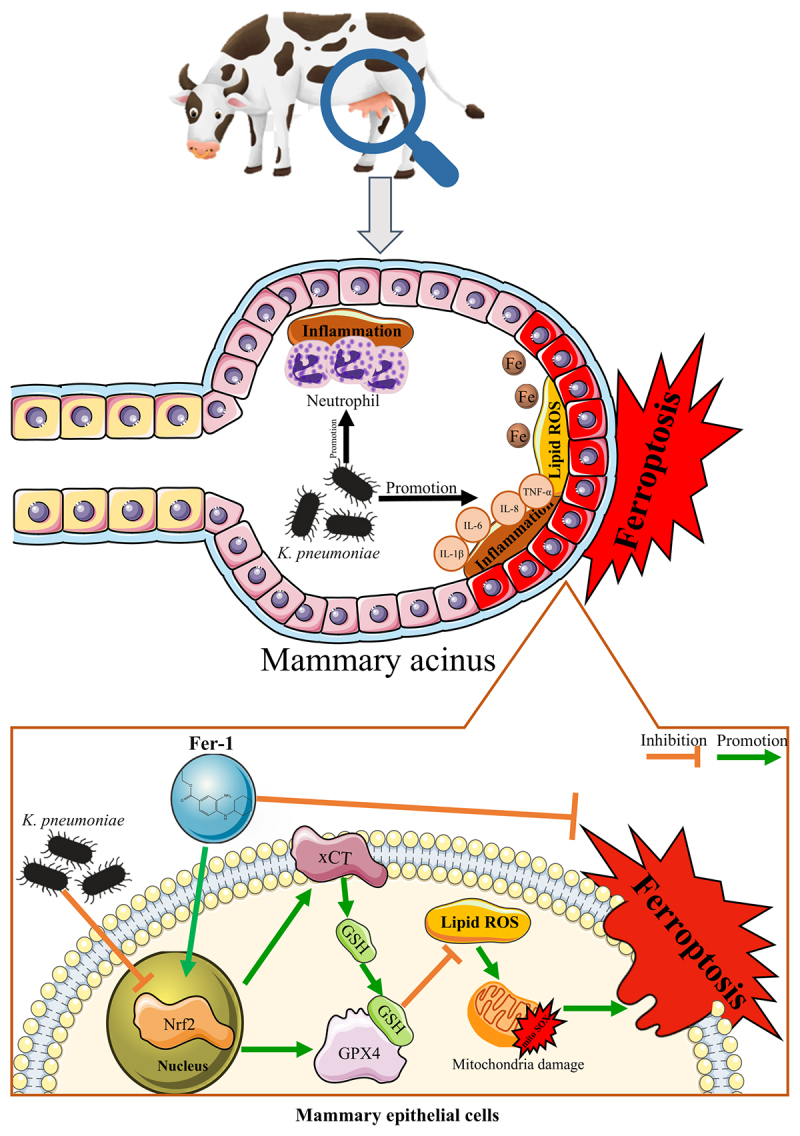
Schematic process of ferroptosis in BMECs infected with *K. pneumoniae*. xCT: cystine/glutamate antiporter; GSH: glutathione; GPX4: glutathione peroxidase 4; Nrf2: nuclear factor erythroid 2-related factor 2; Fe: ferrous ion; IL-1β: interleukin-1β; IL-6: interleukin-6; IL-8: interleukin-8; TNFA-α: tumor necrosis factor-α.

This study established that *K. pneumoniae* infection induced ferroptosis in bovine mammary tissues and epithelial cells by suppressing the Nrf2/xCT/GPX4 axis. Mechanistic investigations revealed that ferroptosis was driven by iron overload, lipid accumulation, and mitochondrial dysfunction, all of which were exacerbated by pathogen-mediated inhibition of Nrf2 nuclear translocation. The ferroptosis inhibitor, Fer-1, demonstrated therapeutic efficacy by reactivating the Nrf2 pathway, thereby restoring redox homeostasis, reducing inflammatory cytokine production, and preserving cellular integrity in infected BMECs (Figure 7). These findings highlight ferroptosis modulation as a promising host-directed strategy to counteract bacterial mastitis, particularly in the context of *K. pneumoniae* infection.

## Data Availability

All data in this article will be obtained through in Mendeley at link: https://doi.org/10.17632/5rhkjgy792.4.
